# The histological type of endometrial cancer is not associated with menopause status at diagnosis

**DOI:** 10.1042/BSR20212192

**Published:** 2022-03-16

**Authors:** Xinyi Sun, Yi Zhang, Fang Shen, Yang Liu, George Qiaoqi Chen, Min Zhao, Qi Chen

**Affiliations:** 1 The Department of Obstetrics and Gynaecology, The University of Auckland, Auckland, New Zealand; 2 Department of Gynaecology, The Hospital of Obstetrics and Gynaecology, Fudan University, Shanghai, China; 3 School of Medicine, The University of Manchester, Manchester, U.K.; 4 Department of Gynaecology, The Affiliated Wuxi Maternity and Child Health Care Hospital of Nanjing Medical University, Wuxi, China

**Keywords:** endometrial cancer, menopause, parity, subtype, survival rate

## Abstract

The latest evidence suggests that type 2 endometrial cancer may not be completely oestrogen-independent, indicating that the status of hormonal change may not be associated with the traditional classification of endometrial cancer, including the histological subtypes. However, this has not been investigated. Menopause is commonly considered a state of hormonal change in women. In the present study, we investigated the association of menopause with the histological types of endometrial cancer. Data on the histological type, menopause status at diagnosis, age at diagnosis, parity, body mass index (BMI), and overall survival rate from 2122 cases were collected. The difference in risk in developing type 1 or type 2 endometrial cancer between premenopausal and postmenopausal patients was 5.457%. A statistical difference in the association of menopause with the histological types between the two groups was seen in endometrioid and serous carcinoma, with a risk difference of 5.6 or 3.8%. A statistical difference in the association of menopause with parity between the groups was only seen in endometrioid and adenosquamous carcinoma, with a risk difference of 7.1 or 3.7%. However, BMI was not associated with histological type and the overall survival rate was not associated with menopause (*P*=0.764). We reported a relatively small difference in the association of menopause with type 1 or type 2, or the histological types of endometrial cancer. The survival rate was not associated with menopause. Our study suggests that menopause status at diagnosis was not strongly associated with the histological subtypes of endometrial cancer.

## Introduction

Endometrial cancer is the third most common cause of death in women with cancer (World Health Organization (WHO) Cancer Report 2014) and annual new cases of endometrial cancer have significantly increased in the last two decades [1]. Endometrial cancer is traditionally classified as type 1 (oestrogen-dependent) and type 2 (oestrogen-independent) based on clinical and endocrine characteristics, which were first described in 1983 [2,3]. However, recent studies indicated that; (1) type 1 and type 2 endometrial cancer share a common pathogenesis and common risk factors [4], for example, a high body mass index (BMI) [5,6], (2) there was no difference in the gonadal hormonal levels between women with type 1 and type 2 endometrial cancer [7], (3) there is no difference in the positivity of oestrogen or progesterone receptor between type 1 and type 2 endometrial cancer [8], and (4) the protective factor in the incidence of endometrial cancer by parity applies to both type 1 and 2 endometrial cancer to a similar degree [9]. All these latest findings clearly suggested that type 2 endometrial cancer may not be completely oestrogen-independent [4], and challenge the traditional classification of endometrial cancer based on endocrine characteristics.

In addition to type 1 and type 2 endometrial cancer, endometrial cancer is also traditionally subtyped into endometrioid carcinoma, adenosquamous carcinoma, serous carcinoma, mucinous carcinoma, and clear-cell carcinoma based on histological characteristics, although some tumours have more than one characteristic [2]. Because the endocrine characteristics cannot solely explain the differences in the pathogenesis between type 1 and type 2 endometrial cancer, this prompted us to question whether these different histological types of endometrial cancer are associated with endocrine characteristics, or whether the histological types of endometrial cancer have different or share the same risk factors. Menopause is commonly considered as a state of endocrine change in a woman’s life. It is well-known that 75–80% of women develop endometrial cancer after menopause in Western countries [10,11], and the median age of diagnosis of endometrial cancer is 63 years [12]. These clinical findings could be due to the decreased levels of progesterone in postmenopausal women, resulting in an increase in the levels of unopposed oestrogen. A recent study reported a positive association between the age of menopause and endometrial cancer suggesting that late menopause increases the risk of developing endometrial cancer [13].

Therefore, to examine whether hormonal changes at menopause associate with the histological types of endometrial cancer, we undertook this retrospective study with large sample size to investigate whether the histological types of endometrial cancer, and overall survival rate for endometrial cancer are associated with menopause status.

## Materials and methods

This retrospective study received approval by the Ethics Committee of Wuxi Maternity and Child Health Hospital, Nanjing Medical University, China and The Hospital of Obstetrics and Gynaecology, Fudan University of China.

### Study population

A total of 2122 women with endometrial cancer from Wuxi Maternity and Child Health Hospital (*n*=1003, from 2008 to 2020) and the Hospital of Obstetrics and Gynaecology of Fudan University (*n*=1119, from 2011 to 2014) were included. Data on age at diagnosis, gravidity, parity, BMI or weight, history of hypertension and diabetes mellitus, and the histological types, and grades of endometrial cancer were collected from the electronic databases from both hospitals. The follow-up survival data were only collected from Wuxi Maternity and Children Health Hospital (*n*=450). The BMI data from Wuxi Maternity and Child Health Hospital were only collected from 2016, as weight was collected between 2008 and 2015. Menopause status at time of diagnosis and a history of hypertension or diabetes mellitus were self-reported.

Endometrial carcinomas are histologically classified into five types including endometrioid carcinoma, adenosquamous carcinoma, serous carcinoma, mucinous carcinoma, and clear-cell carcinoma, according to the WHO classification guideline of gynaecologic cancer [14]. On the basis of the histological types and the grades, type 1 endometrial cancer includes endometrioid carcinoma with grade 1 or 2, and adenosquamous carcinoma and mucinous carcinoma. Type 2 endometrial cancer includes serous carcinoma, and clear-cell carcinoma and endometrioid carcinoma with grade 3, according to the classification of the International Federation of Gynaecology and Obstetrics (FIGO).

### Statistical analysis

Data on clinical parameters were expressed by median and range, or percentage. The statistical analysis on the association of menopause with type 1 or type 2 endometrial cancer, or the histological types of endometrial cancer, as well as the association of parity with the histological types of endometrial cancer, were assessed by Chi-square test using the SPSS software package (version 26, SPSS Inc., Chicago, IL, U.S.A.). The risk difference was assessed using OpenEpi Software (version 3.03). The statistical difference on the association of overall survival rate and menopause status was assessed by the Cox regression using SPSS software package. The difference in the years of onset after menopause between histologic types of endometrial cancer was assessed by an ANOVA (nonparametric test) using the SPSS software. *P*<0.05 was considered statistically significant.

## Results

### Clinical parameters of the study cohort

Clinical parameters for women with endometrial cancer are summarised in Table 1. The median age at diagnosis was 55 years (range from 20 to 88 years) in women with endometrial cancer. A total of 1126 (53%) women had more than two live births. Of the 2122 women with endometrial cancer, 1779 (83.8%) were diagnosed with endometrioid carcinoma and 139 (6.6%) women were diagnosed with adenosquamous carcinoma; 96 (4.5%) women were diagnosed with serous carcinoma and 52 (2.5%) women were diagnosed with mucinous carcinoma; 56 (2.6%) women were diagnosed with clear-cell carcinoma; 858 (40%) of women were diagnosed before menopause.

**Table 1:** Clinical parameters of study population

	Endometrial cancer (*n*=2122)
Age at diagnosis (years, median/range)	55 (20–88)
Parity (≥2) (number, %)	1126 (53%)
Histological types (number, %)	
Endometrioid carcinoma	1779 (83.8%)
Adenosquamous carcinoma	139 (6.6%)
Serous carcinoma	96 (4.5%)
Mucinous carcinoma	52 (2.5%)
Clear-cell carcinoma	56 (2.6%)
Premenopause (number, %)	858 (40.4%)
Post menopause (number, %)	1264 (59.6%)

### The association of menopause with subtypes of endometrial cancer

We first analysed the association of menopause with the subtypes (type 1 or type 2) of endometrial cancer (Table 2). The proportion of type 1 endometrial cancer in premenopausal patients was significantly higher than in postmenopausal patients (93 vs 88%, *P*<0.0001). In contrast, the proportion of type 2 endometrial cancer in postmenopausal patients was significantly higher than in premenopausal patients (12 vs 7%, *P*<0.0001). The risk difference between the two groups was 5.457% (95% confidence interval (CI): 3.035, 7.878).

**Table 2:** The association of menopause with subtypes of endometrial cancer

	Premenopause (*n*=858)	Postmenopause (*n*=1264)	Risk difference (95% CI)
Type 1	803 (93%)	1114 (88%)	5.457% (3.035, 7.878)^*^
Type 2	55 (7%)	150 (12%)	

* *P*<0.0001.

### The association of menopause with the histological types of endometrial cancer

We then analysed the association of menopause with the histological types of endometrial cancer (Table 3). The proportion of patients with endometrioid carcinoma in premenopausal patients was significantly higher than in postmenopausal patients (87 vs 81%, *P*<0.0001). The risk difference between the two groups was 5.613% (95% CI: 2.519, 8.707). The proportion of serous carcinoma in postmenopausal patients was significantly higher than in premenopausal patients (6.0 vs 2.2%, *P*<0.0001). The risk difference between the two groups was 3.877% (95% CI: 2.232, 5.523). However, there was no significant difference in the proportion of adenosquamous carcinoma, mucinous carcinoma, or clear-cell carcinoma between premenopausal and postmenopausal patients (Table 3, *P*>0.06). In addition, we found that in postmenopausal patients, patients with serous carcinoma had a significant delay in the time of onset of cancer, compared with patients with other histological types of endometrial cancer (Table 4, 11 years vs 7 or 8.5 or 10 years, *P*<0.0001). There was no difference in the time of onset of endometrial cancer after menopause among patients with other histological types of endometrial cancer.

**Table 3:** The association of menopause with histological subtypes of endometrial cancer

	Premenopause (*n*=858)	Postmenopause (*n*=1264)	Risk difference (95% CI)
Endometrioid carcinoma (*n*=1779)	748 (87%)	1031 (81%)	**5.613% (2.519, 8.707)** ^*^
Adenosquamous carcinoma (*n*=139)	59 (6.8%)	80 (6.4%)	0.547% (−1.613, 2.708)
Serous carcinoma (*n*=96)	19 (2.2%)	77 (6.0%)	**3.877% (2.232, 5.523)** ^*^
Mucinous carcinoma (*n*=52)	14 (1.6%)	38 (3.0%)	1.375% (0.108, 2.641)
Clear-cell carcinoma (*n*=56)	18 (2.1%)	38 (3.0%)	0.9084% (0.4353, 2.252)

* *P*<0.0001. Bold values signify the statistical difference of risk difference.

**Table 4:** Years of diagnosis with endometrial cancer after menopause in postmenopausal women

	Years of diagnosis after menopause (years, median/range)	*P*-value (ANOVA test)
Endometrioid carcinoma (*n*=1031)	7 (0–43)	*P*<0.0001
Adenosquamous carcinoma (*n*=78)	8.5 (0–30)	
Serous carcinoma (*n*=75)	**11 (3–30)**	
Mucinous carcinoma (*n*=38)	7 (1–34)	
Clear-cell carcinoma (*n*=38)	10 (2–30)	

Bold values signify that only serous carcinoma had a statistically significant delay in the time of onset of cancer, compared with patients with other histological types of endometrial cancer.

### The association of parity with histological types of endometrial cancer

Due to the One-Child policy instituted in China until October 2015, the majority of patients had one to three live births. We then divided patients with parity < 2, and patients with parity ≥ 2 (Table 5). The proportion of endometrioid carcinoma in patients with parity ≥ 2 was significantly higher than in women with parity <2 (87 vs 80%, *P*<0.0001). The risk difference between the two groups was 7.191% (95% CI: 4.034, 10.35). The proportion of adenosquamous carcinoma in women with parity ≥ 2 was higher than in patients with parity < 2 (8.5 vs 4.8%, *P*<0.0001). The risk difference between the two groups was 3.78% (95% CI: 1.601, 5.876). However, there was no significant difference in the proportion of serous carcinoma, mucinous carcinoma, or clear-cell carcinoma between patients with parity < 2 and patients with parity ≥ 2 (*P*>0.05).

**Table 5:** The association of parity with histological types of endometrial cancer

	Parity (<2) (*n*=996)	Parity (≥2) (*n*=1126)	Risk difference (95% CI)
Endometrioid carcinoma (number, %)	797 (80%)	982 (87%)	7.191% (4.034, 10.35)^*^
Adenosquamous carcinoma (number, %)	85 (8.5%)	54 (4.8%)	3.738% (1.601, 5.876)^*^
Serous carcinoma (number, %)	49 (4.9%)	47 (4.1%)	0.7195% (−1.065, 2.504)
Mucinous carcinoma (number, %)	31 (3.1%)	21 (1.8%)	1.247% (−0.0894,2.584)
Clear-cell carcinoma (number, %)	34 (3.4%)	22 (2.0%)	1.46% (0.0724, 2.847)

* *P*<0.0001. Bold values signify the statistical difference of risk difference.

### The association of BMI with the histological types of endometrial cancer

As obesity is one of the main risk factors for developing endometrial cancer [15], we then analysed the association of BMI with specific histological types of endometrial cancer. Due to the small mucinous carcinoma and clear-cell carcinoma sample sizes, the analysis of BMI with these two histological types was excluded. Overall, there was no association of BMI with the three histological types of endometrial cancer, with the exception of a significantly lower incidence of serous carcinoma in obese women (12 vs 23% in endometrial carcinoma or 20% in adenosquamous carcinoma, *P*=0.005).

We then analysed the association of BMI with each histological type of endometrial cancer. Compared with patients with normal weights, BMI was not associated with any of the three histological types of endometrial cancer (Table 6, *P*>0.05), with the exception of underweight patients with serous carcinoma. The odds ratio for developing serous carcinoma in patients who were underweight was 15.46 (95% CI: 7.671, 30.15, *P*<0.0001), compared with patients who had a normal weight.

**Table 6:** The association of BMI with histological types of endometrial cancer

	Underweight (*n*=33)	Normal weight (*n*=428)	Overweight (*n*=707)	Obese (*n*=328)
Endometrioid carcinoma (*n*=1329)	25 (75%)	378 (88%)	625 (88%)	301 (92%)
OR (95% CI)	2.419 (1.093, 5.486)	**Referent**	0.991 (0.683, 1.454)	0.678 (0.421, 1.09)
Adenosquamous carcinoma (*n*=77)	1 (3%)	24 (5.6%)	36 (5.1%)	16 (4.8%)
OR (95% CI)	1.901 (0.325, 20.25)	**Referent**	1.107 (0.68, 1.891)	1.158 (0.611, 2.26)
Serous Carcinoma (n = 90)	7 (21%)	26 (6%)	46 (6.5%)	11 (3.3%)
OR (95% CI)	**15.46 (7.671, 30.15)**	**Referent**	0.936 (0.572, 1.533)	1.834 (0.884, 3.801)

BMI data from 2008 to 2015 from Wuxi Maternity and Children Hospital was not available, due to the weight collection in that period.

The odds ratio (OR) was referent to patients with normal weight.

Bold values signify statistically significance.

### The association of the status of menopause with overall survival rate

**Figure 1:**
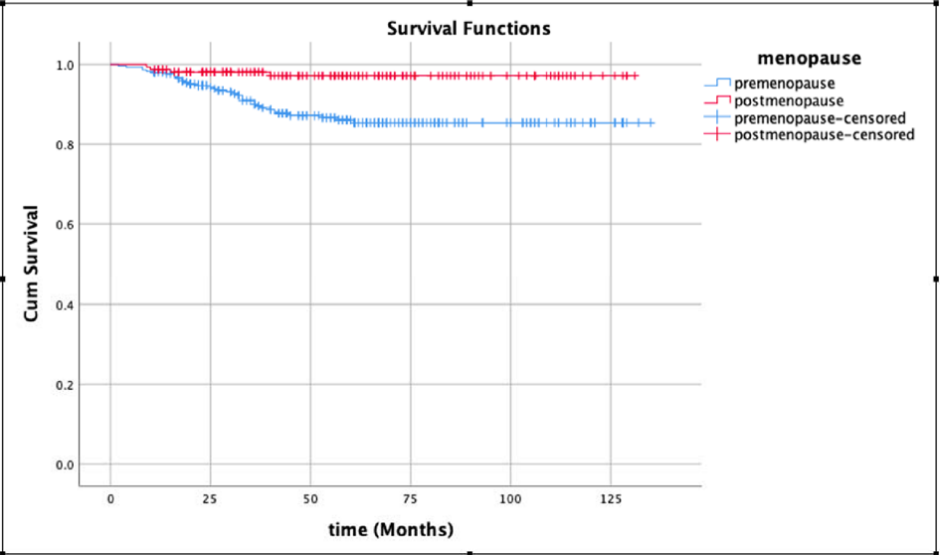
The overall survival chart

Due to data availability, we could only analyse overall survival rate from one hospital database (Wuxi Maternity and Child Health Hospital). As shown in Figure 1, after adjusting for age at diagnosis and the grade of endometrial cancer, the overall survival rate was not statistically different between premenopausal and postmenopausal patients (*P*=0.764).

## Discussion

In this retrospective study, we found that the proportion of type 1 or type 2 endometrial cancer was statistically significantly different between premenopausal and postmenopausal patients. An association of menopause with the histological types of endometrial cancer was only seen for endometrioid carcinoma and adenosquamous carcinoma, but not for the other three histological types. The number of gravidities was associated with endometrioid carcinoma and adenosquamous carcinoma and overall BMI was not associated with the histological types of endometrial cancer. However, the overall survival rate was not associated with menopause status at the time of diagnosis.

Endometrial cancer is an endocrine-related cancer and is traditionally subdivided into type 1 oestrogen-dependent and type 2 oestrogen-independent cancer, on the basis of its endocrine characteristics. Menopause is a status of hormonal change in a woman’s life and most endometrial cancer occurs after menopause. A recent study reported an association of age at menopause with endometrial cancer [13]. However, whether the changes in endocrine characteristics associated with oestrogen-dependent subtypes of endometrial cancer has not been fully investigated. In our current study, we confirmed 60% of cases of endometrial cancer were in postmenopausal women. We also found a higher number of premenopausal patients with type 1 endometrial cancer. In contrast, a higher number of patients with type 2 endometrial cancer were postmenopause. Previous studies reported that type 1 endometrial cancer normally expresses high levels of oestrogen receptor α (ER) and is thought to be hormonally driven (reviewed in [16]), while type 2 endometrial cancer is less likely to express ER [17]. However, our recent study found no difference in the positivity of ER or progesterone receptor (PR) between type 1 and type 2 endometrial cancer [8]. In our current study, we found that the risk difference of developing type 1 or type 2 endometrial cancer between premenopausal and postmenopausal women was 5.5%. This relatively small difference suggests that the association of menopause with subtypes (type 1 and type 2) of endometrial cancer may not be clinically important. Our data further confirmed the findings from a previous study indicating type 2 endometrial cancer may not be completely oestrogen-independent [4].

To date, the association of hormonal change with the histological types of endometrial cancer has not been well-investigated. In our current study, we found a significant association between menopause status at the time of diagnosis and endometrioid or serous carcinoma, but not the other histological types of endometrial cancer. In addition, years of diagnosis of endometrial cancer after menopause was not different among the histological types, except for serous carcinoma. As the risk difference of developing endometrioid carcinoma or serous carcinoma between premenopausal and postmenopausal women was relatively small (5.6 or 3.8%, respectively). This suggests that the histological types of endometrial cancer may not be associated with menopause status or the changes in gonadal hormonal levels that go along with this.

A previous study found a difference in risk factors such as BMI, parity, and age of menarche between the histological types of endometrial cancer. BMI, parity, and age of menarche were significantly associated with endometrioid carcinoma, but not with serous carcinoma [18]. In addition, the serum levels of endogenous oestrogen and sex hormone binding globulin (SHBG) were different between patients with endometrioid carcinoma and patients with serous carcinoma, after adjusting for age and BMI [18]. The differences in the risk factors or sex hormone levels between different histological types of endometrial cancer were explained by how endometrioid adenocarcinoma develops from endometrial hyperplasia, while serous carcinoma develops from atrophic rather than hyperplastic epithelium [19]. This difference suggests that the development of endometrioid adenocarcinoma requires a setting of excess oestrogen exposure. However, other minor histological types of endometrial cancer, such as serous carcinoma do not seem to be related to oestrogenic risk factors or elevated serum hormone levels [19].

The negative association of parity and the incidence of endometrial cancer has been well reported. This negative association was also applicable to the individual histological type of endometrial cancer, except for clear-cell carcinoma [4]. Due to the One-Child policy instituted in China until October 2015, the majority of patients had one to three live births. When we divided patients with less than two live births from those with two or more live births, we found the incidence of developing serous carcinoma, mucinous carcinoma, or clear-cell carcinoma was not associated with the number of live births. In contrast, the incidence of developing endometrioid carcinoma or adenosquamous carcinoma was significanlty associated with parity. Patients with two or more live births have an increased incidence of developing endometrioid carcinoma, while patients with less than two live births have an increased incidence of developing adenosquamous carcinoma. However, the risk difference in developing endometrioid carcinoma or adenosquamous carcinoma between patients with less than two and two or more live births was relatively small (7.1 or 3.7% respectively), and whether this has a clinical application needs to be further studied.

Obesity significantly contributes to the development of endometrial cancer [15]. Although a previous study reported a positive trend association of BMI with individual histological type of endometrial cancer [4], the association of BMI with specific histological types of endometrial cancer has not been fully reported. However, developing endometrioid carcinoma seems more likely to require a setting of excess oestrogen exposure, while the other histological types do not [19]. It is well-known that body fat is one of the main sources of oestrogen production [20]. However, this may not be the case [4]. In our current study, we found that while there was no association of BMI with the three histological types (endometrioid carcinoma, adenosquamous carcinoma and serous carcinoma) of endometrial cancer, our data showed a significantly lower incidence of serous carcinoma in obese patients. In addition, when we analysed the association of BMI with individual histological types of endometrial cancer, we found no association of BMI, with the exception of underweight patients who had an increase in the odds ratio for developing serous carcinoma (15.46; 95% CI: 7.671, 30.15, *P*<0.0001), compared with patients with serous carcinoma who were normal weight. This could be due to the small sample size in underweight patients with serous carcinoma, and further work is required to confirm our finding. Taken together our data suggests that developing specific histological types of endometrial cancer was not correlated to BMI.

The prognosis of type 1 and type 2 endometrial cancer is significantly different, with type 1 endometrial cancer showing a favourable outcome. Although the overall 5-year relative survival rate is over 80% worldwide [21], whether menopause status at the time of endometrial cancer diagnosis is associated with the overall survival rate had not yet been investigated. In our current study, we further found that, after adjusting for the age and stage of cancer, the overall survival rate was not associated with the menopause status at the time of diagnosis. Our data may suggest that the overall survival of endometrial cancer is not associated with the changes in sex hormone levels.

There are some limitations in the present study. The status of menopause was self-reported, which may cause a bias. There was a relatively small sample size in mucinous carcinoma and clear-cell carcinoma, in particular when these cases were subdivided into BMI categories.

## Conclusion

Increasing evidence suggested that the traditional distinction of type 1 and type 2 endometrial cancer should be reconsidered [2,4]. Here, our data demonstrate that the incidence of histological subtypes of endometrial cancer and the overall survival rate are similar between premenopausal and postmenopausal women. In addition, BMI and parity were also not strongly associated with any of the different histological subtypes of endometrial cancer. Our study suggests that menopause status at diagnosis, reflecting sex hormonal changes in women, was not strongly associated with histological subtypes of endometrial cancer.

## Data Availability

The datasets used and/or analyzed during the current study available from the corresponding author on reasonable request.

## Open Access

Open access for this article was enabled by the participation of The University of Auckland in an all-inclusive *Read & Publish* agreement with Portland Press and the Biochemical Society under a transformative agreement with CAUL.

## Abbreviations

BMI: body mass index
CI: confidence interval
ER: oestrogen receptor
WHO: World Health Organization
